# EAU guidelines for management of penile cancer

**DOI:** 10.4103/0970-1591.30277

**Published:** 2007

**Authors:** Paul K. Hegarty

**Affiliations:** Institute of Urology, University College London Hospital, United Kingdom

Dear Sir,

I was impressed to see the attention the Journal[1,2] pays to penile cancer. We have been using the European Association of Urology (EAU) guidelines for several years. The guidelines recommend prophylactic inguinal lymphadenectomy based on the stage and grade of the primary tumor. Patients with impalpable inguinal lymph nodes are categorized into low, intermediate- and high-risk groups. Cases in the intermediate risk group with lymphovascular invasion or with growth patterns that indicate aggressive disease are offered lymphadenectomy. These categories have been based upon retrospective studies. Our recent prospective data of 100 consecutive cases has shown the value of the guidelines.

Firstly, no patient in the low-risk group who had surveillance rather than inguinal node dissection, developed regional or distant metastases. Thus it is appropriate for these patients to avoid prophylactic surgery.

Of the patients for whom prophylactic dissection was prescribed, only 18% had micrometastatic disease, thus 72% were overtreated. All patients who were free of nodal metastases (N0) were cured. This serves as a great psychological relief to patients as soon as the results of histopathology return. Patients with only one superficial inguinal node involved (N1) had a 100% survival, implying that lymphadenectomy is curative in these men. N2 and N3 disease had progressively poorer survival, but still many were cured by surgery alone.

The risk of nodal involvement and death was predicted well by grade of the primary tumor, whereas the T stage was not helpful. Thus basing the criteria for prophylactic dissection on the current T stage is flawed and needs to be addressed.

Overall the EAU guidelines are helpful as they promote curative surgery albeit exposing some patients to needless morbidity. Better prognostic indicators should help in categorizing patients in the future.
